# Higher Intron Retention Levels in Female Alzheimer's Brains May Be Linked to Disease Prevalence

**DOI:** 10.1111/acel.14457

**Published:** 2025-01-13

**Authors:** Ching‐Thong Choo, Chao‐Yong Leow, Chin‐Tong Ong

**Affiliations:** ^1^ Temasek Life Sciences Laboratory Singapore Singapore; ^2^ Department of Biological Sciences National University of Singapore Singapore Singapore

**Keywords:** Alzheimer's disease, CTCF, H3K27ac, intron retention, proteomics, RBP, sex‐specific

## Abstract

Multimodal study of Alzheimer's disease (AD) dorsolateral prefrontal cortex (DLPFC) showed AD‐related aberrant intron retention (IR) and proteomic changes not observed at the RNA level. However, the role of sex and how IR may impact the proteome are unclear. Analysis of DLPFC transcriptome showed a clear sex‐biased pattern where female AD had 1645 elevated IR events compared to 80 in male AD DLPFC. Increased IR is correlated with lower mRNA levels, suggestive of nonsense‐mediated mRNA decay. Two hundred thirty‐three mRNAs with elevated IR in females were curated AD genes enriched for ubiquitin‐like protein ligase and Tau protein binding. Increased IR genes in combined sex and female AD cohorts showed significant changes in their protein expression patterns with 11%–24% of them differential expressed proteins (DEP), alluding to the regulation of AD proteome by IR independent of RNA level. Upregulated DEPs in male AD were linked to RNA splicing that may prevent aberrant IR, whereas in female AD, they overlapped significantly more with the MAPK/metabolism module associated with cognitive decline. IR genes appeared to be significantly downregulated in specific female AD inhibitory and excitatory neurons compared to control. Differentially retained introns in female AD have elevated H3K27ac marks, strong CTCF binding at their flanking exons, and enriched for PABPC1 motif. Given that H3K27ac is repressive over gene bodies in aged brain and CTCF impedes transcription elongation, their binding patterns can delay co‐transcriptional recruitment of spliceosome to cause IR, which may in turn contribute to different trajectories of AD pathology in women.

## Graphical abstract

Enrichment of H3K27ac at introns with CTCF binding at their flanking exons may impair co‐transcriptional recruitment of spliceosome, leading to increased intron retention (IR) in female AD DLPFC. Male AD DLPFC has elevated protein levels of spliceosome that may protect against aberrant IR. Differential IR genes might in turn change the expression levels of mRNA and proteins in female AD DLPFC compared to healthy control.

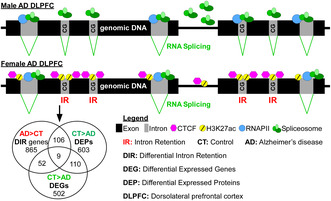

## 1 Introduction, Results, and Discussion

**FIGURE 1:**
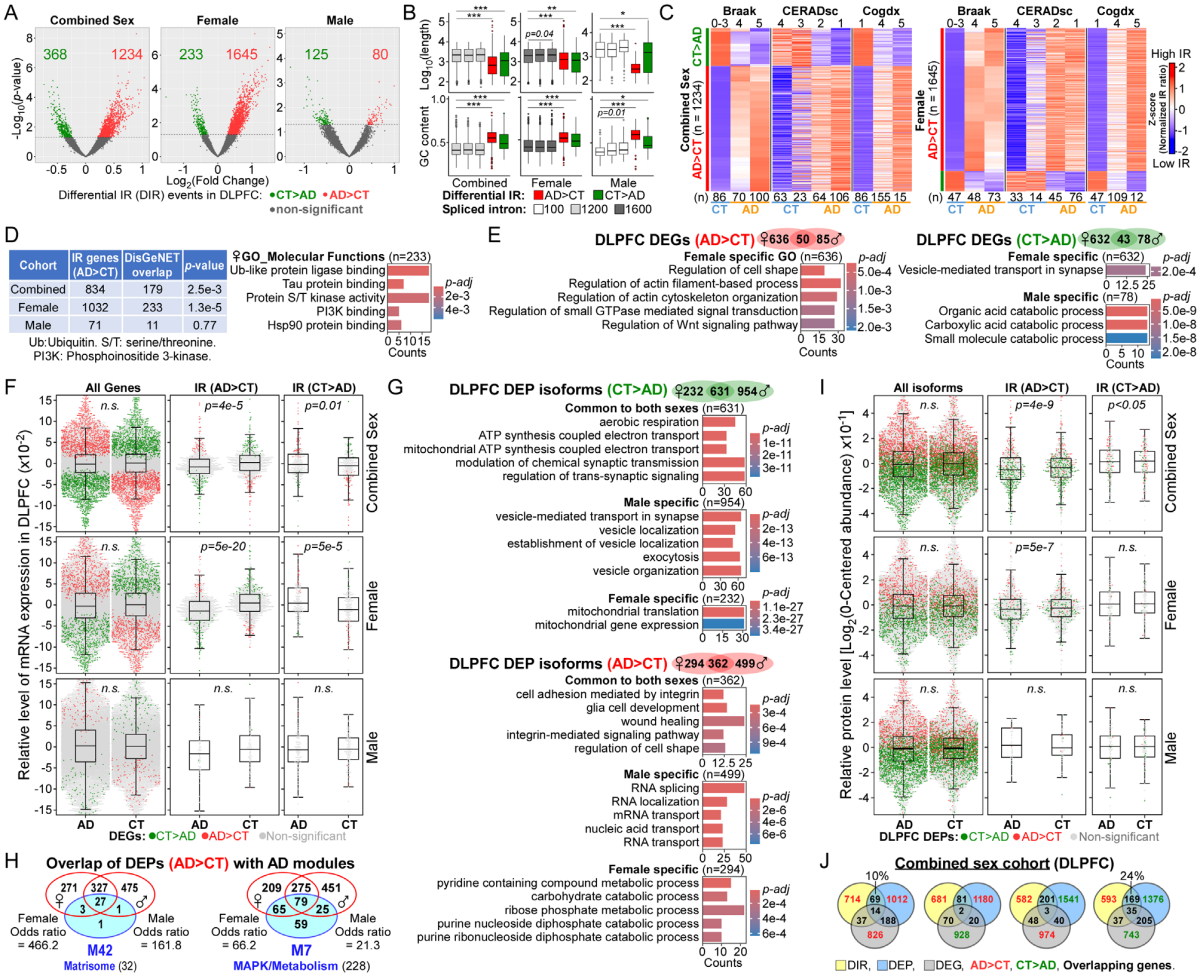
Elevated IR in female AD DLPFC altered RNA and protein patterns. (A) Volcano plots of DIR events between CT and AD. (B) Boxplots for (*top*) intron length and (*bottom*) GC content of random‐picked spliced and differential retained introns. *p < 0.05; **p = 6.2e−12; ***p < 2.2e−16 with Wilcoxon rank sum test and continuity correction. (C) Heatmap of IR levels stratified by Braak, CERAD, and cognitive diagnosis (Cogdx) scores in combined sex and female cohorts. (*n*) is the number of individuals per group. (D) (*Left*) Overlap of curated AD genes with DIR genes from different cohorts. *p*‐value was computed by Pearson's *χ* ^2^‐test and Yates' continuity correction. (*Right*) GO terms of overlapped female IR genes. (E) Top GO terms of sex‐specific DEGs between CT and AD. (F) Beeswarm boxplots showing expression patterns of all or DIR genes between CT and AD with *p*‐value computed by paired two‐tailed *t*‐test. (G) GO terms of common and sex‐specific DEPs between CT and AD. (H) Overlap of AD > CT DEPs (red) from each sex with AD protein modules (blue). Parentheses show the number of proteins in each module. (I) Beeswarm boxplots showing protein levels of all or DIR genes between CT and AD with *p*‐values computed by paired two‐tailed *t*‐test. (J) Venn diagrams of the numbers and percentage of DIR genes (AD > CT) that overlapped with DEGs or DEPs between CT and AD.

Pathogenesis and dysfunction of DLPFC occur at different AD stages with proteomic changes not observed at the RNA level (Johnson et al. 2022; Kumar et al. 2017). While aberrant IR is linked to AD (Adusumalli et al. 2019; Raj et al. 2018), the effect of sex and how IR may impact the proteome are unclear. Analysis of DLPFC mRNAs from the entire cohort revealed 1234 elevated differential IR (DIR) events in AD as compared to control (CT) with 368 DIR in reverse trend (Figure 1A and Data SD2). Unlike low DIR in males, there was approximately sevenfold more increased IR events in female AD than in CT (1645 vs. 233) (Figure 1A). IR was validated by qPCR of brain mRNA (Figure S1A). Like other cells (Braunschweig et al. 2014), retained introns in AD have shorter length and higher GC content (Figure 1B and Figure S1B). Stratification of AD traits in combined sex showed changing IR levels across Braak stages (Tau), CERAD (Amyloid‐β), and Cogdx (cognitive) scores, suggestive of its link to increasing Tau/Αβ loads and cognitive decline in AD (Figure 1C). IR affected unique genes in a sex‐specific manner depending on AD traits but correlated poorly with Braak stages in CT (Figure 1C and Figure S1C,D). Two hundred thirty‐three female DIR genes were curated AD genes linked to ubiquitin‐like protein ligase and Tau protein binding, alluding to its causative roles in AD pathology (Figure 1D and Data SD3).

We next identified differentially expressed genes (DEGs) after data normalization and regression. The combined sex cohort showed 1066 up‐ and 1020 downregulated DEGs in AD compared to CT (Data SD4). In female AD, there were 686 up‐ and 675 down‐regulated DEGs compared to CT. In line with fewer DEGs in males (Guo et al. 2023), there were only 135 up‐ and 121 down‐regulated DEGs in male AD versus CT with unique pathways affected in each sex (Figure 1E and Data SD5). In contrast to all or random spliced genes, there were significant differences in the expression pattern of DIR genes between CT and AD, alluding to nonsense‐mediated mRNA decay (NMD) (Figure 1F, Figure S1E and Data SD5C). Poor correlation of DIR and DEGs in male is likely due to their low numbers.

As RNA splicing may affect protein translation, we asked if IR alters the proteome. Combined sex cohort showed 1335 up‐ and 1907 down‐regulated differential expressed protein (DEP) isoforms in AD as compared to CT DLPFC (Data SD6). Similar to sex‐biased protein expression in DLPFC (Wingo et al. 2023), there were 861 up‐ and 1585 down‐regulated isoforms in male AD as compared to CT but lower numbers in female (656 up and 863 down DEPs in AD vs. CT). In male AD, upregulated DEPs linked to RNA splicing may block aberrant IR (Figure 1G and Data SD7). Compared to males, upregulated DEPs in female AD overlapped significantly more with the AD‐related M7 protein module which is a prime effector of cognitive decline (Figure 1H and Data SD6) (Johnson et al. 2022). In contrast to all or random spliced genes, DIR (AD > CT) genes in combined sex and female cohorts have significant changes in their protein expression patterns between CT and AD (Figure 1I, Figure S1F and Data SD6) with 11%–24% of them DEPs involved in synaptic and transport biology (Figure 1J, Figure S1G,H and Data SD7). This correlative result suggests that IR may impact the proteome in AD DLPFC without altering RNA.

**FIGURE 2:**
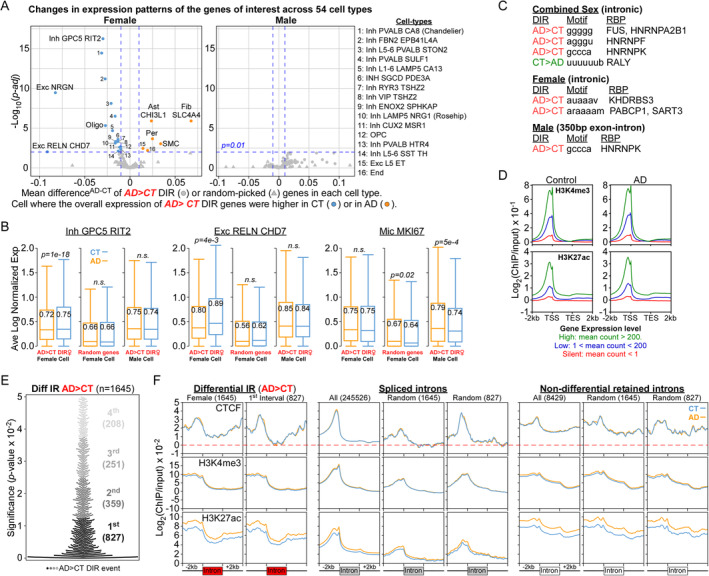
Distinct cell types and epigenetic features for female DIR genes. (A) Volcano plot showing mean difference in mRNA expression of DIR (O) or random‐picked genes (Δ) between CT and AD in 54 cell types derived from female or male PFC. Each point is a unique cell. Inh GPC5 RIT2 and Exc RELN CHD7 cells were chosen based on their adjusted *p*‐value (Benjamini–Hochberg) and mean difference (AD‐CT). (B) Boxplots showing downregulation of female DIR genes in AD Inh GPC5 RIT2 and Exc RELN CHD7 cells as compared to CT. This is not observed in Mic MKI67, male, or for random‐picked genes. Number shows the average log normalized expression with *p*‐value computed by paired *t*‐test. (C) Unique RBP motifs at the differentially retained introns and exon‐intron junctions from different cohorts. (D) Metaplots of H3K4me3 and H3K27ac over genes with different expression levels. (E) Female IR events were divided into four intervals based on their *p*‐values. (F) Metaplots of CTCF, H3K4me3, and H3K27ac over different introns and their flanking sequences in female CT and AD.

The high concordance between bulk and snRNA‐seq DEGs led us to survey DIR genes in 54 cell types from CT and AD PFC (Figure S2A) (Mathys et al. 2023). Expression level of female AD > CT DIR genes in specific inhibitory and excitatory neurons (Inh GPC5 RIT2 and Exc RELN CHD7) was significantly higher in female CT than AD (Figure 2A and Data SD8). Such difference was not observed with other genes, in males or cells like microglia MKI67 (Figure 2B and Figure S2B,C). As IR can alter mRNA levels, this data suggests that DIR may have occurred in these neuro‐subtypes.

Histone marks, zinc finger transcription factor (ZNF) and RNA binding proteins (RBP) can regulate IR (Monteuuis et al. 2019; Ullah et al. 2023). Retained introns in female AD were bound by RBP like SART3, which recycles spliceosome (Ayers et al. 2023), and PABPC1, which may mis‐localize to Tau11i aggregates caused by IR in female AD (Figure 2C and Data SD9) (Lester et al. 2021; Ngian et al. 2022).

H3K4me3 and H3K27ac bound to −1 kb region of TSS of highly expressed genes, with H3K27ac extended into gene bodies in DLPFC (Figure 2D). Retained introns in female AD were also enriched for ZNF motifs like CTCF which regulates chromatin looping with cohesin and RNA splicing (Figure S3A,B and Data SD9) (Alharbi et al. 2021). CTCF bound to TSS with lower signal around differential peaks in female AD DLPFC (Figure S3C) (Patel, Ren, and Yan 2023). We mapped CTCF occupancy and histone marks at top 827 and all introns with elevated IR in female AD (Figure 2E). Unlike spliced and non‐differential retained introns, CTCF bound strongly to the flanking exons of differentially retained introns in AD (Figure 2F and Figure S3D) where it may affect splicing by chromatin looping or stalling RNAPII at the exons. Studying how cohesin regulates IR will provide insights into their roles in AD pathology.

H3K4me3 and H3K27ac levels were elevated in AD brains (Cao et al. 2020; Nativio et al. 2020). Unlike their profiles at non‐differential retained and spliced introns, both marks localized to −2 kb upstream of differentially retained introns with sharp drop at exon–intron junction (Figure 2F and Figure S3D). As H3K27ac was highly repressive over gene bodies (Cheng et al. 2018), the high H3K27ac levels observed over DIR genes in AD DLPFC may block co‐transcriptional recruitment of spliceosome to cause IR.

Circular RNA made from back‐splicing can act as an miRNA sponge that regulates AD pathology (Dube et al. 2019) while sex‐specific decline of cholinergic‐targeting tRNA fragments drove cholinergic dysfunction in female AD (Shulman et al. 2023). RNA splicing is disrupted by Tau aggregate (Raj et al. 2018) with aberrant IR also seen in other dementia (Ngian et al. 2022). Future works will need to study the mechanism by which IR affects the proteome in AD and other tauopathies.

## 2 Method

Datasets and methods are in S1.

## Author Contributions

Chin‐Tong Ong and Ching‐Thong Choo: experimental design, data analyses, and paper writing. Chao‐Yong Leow: qPCR.

## Conflicts of Interest

The authors declare no conflicts of interest.

## Supporting information


Appendix S1

## Data Availability

No new dataset was generated.
