# Supplementary material for: Higher Intron Retention Levels in Female Alzheimer's Brains May Be Linked to Disease Prevalence
Source: Aging Cell. 2025 Jan 13;24(2):e14457. doi: 10.1111/acel.14457 (PMC11822637; doi:10.1111/acel.14457)
Supplement: Supplementary file 1 — Appendix S1 [file ACEL-24-e14457-s001.pdf]

Supplemental Information

CONTENT

1. Supplemental Figure Fig.S1 to Fig.S3 and their legends.
2. Supplemental Methods.
3. Description of Supplemental Data SD1 to SD9.

https://drive.google.com/drive/folders/1GfHERCVCQ8iiOT7DKREetnCfH8G3PwOX?usp=sharing

4. References

SUPPLEMENTAL FIGURE

Figure S1

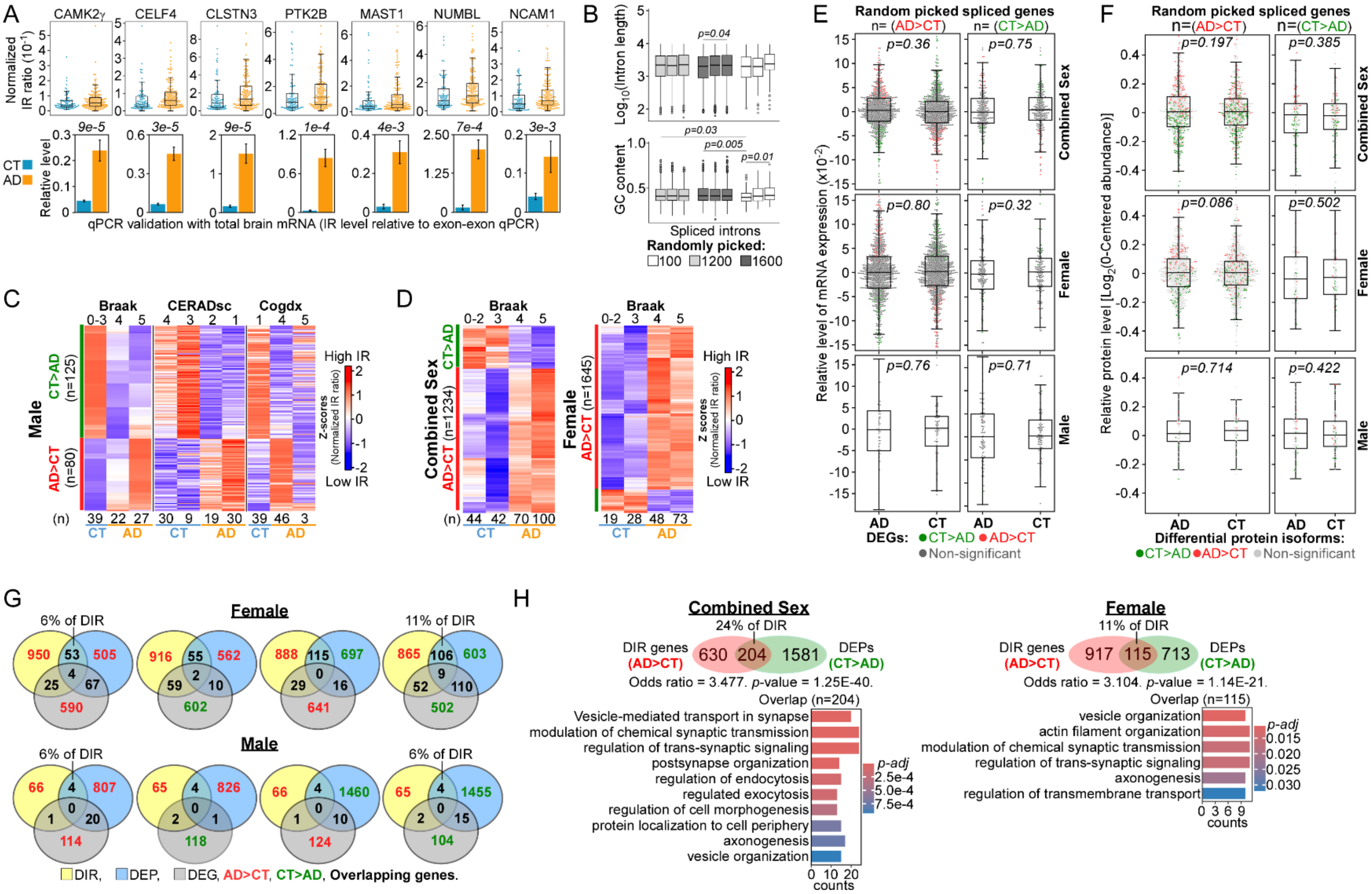

A) (*Top*) Boxplots of selected differential retained introns between healthy CT (blue) and AD patients (orange) where each dot represents an individual human subject. (*Bottom*) qPCR validation of specific retained introns using site-specific primers on cDNA prepared from human brain total RNA. Data are presented as mean ± S.D. (qPCR triplicates, 2-tailed t-test). Related to Figure 1A.

B) (*Top*) Boxplot showing the distribution of intronic length and (*bottom*) GC content of different numbers of random-picked spliced introns. The number of random-picked genes "n" matched the number of DIR genes from various categories (sex, AD>CT or CT>AD). Wilcoxon rank sum test with continuity correction. Only significant p-values were shown. Related to Figure 1B.

C) Expression heatmap of differentially retained introns stratified by Braak stages, CERAD score (sc) and cognitive diagnosis (Cogdx) in male cohort. "n" is the number of individuals in

each group. CT>AD and AD>CT DIR events were indicated by “green” and “red” bar respectively. Related to Figure 1C.

D) Expression heatmap of differentially retained introns stratified by Braak stages in combined sex and female cohorts. “n” is the number of individuals in each group. Related to Figure 1C.

E) Beeswarm boxplots illustrating the relative mRNA expression of random-picked genes with spliced introns. The number of random-picked genes “n” matched the number of DIR genes from various categories (sex, AD>CT or CT>AD). Paired two-tailed student’s *t*-test. Related to Figure 1F.

F) Beeswarm boxplots illustrating the relative protein expression of random-picked genes with spliced introns. The number of random-picked genes “n” matched the number of DIR genes from various categories. Paired two-tailed student’s *t*-test. Related to Figure 1I.

G) Venn diagrams of DIR genes (AD>CT) from (*top*) female and (*bottom*) male cohorts that overlapped with DEGs and DEPs. Note that some genes were lost in the overlap due to the absence of official gene symbol. Related to Figure 1J.

H) (*Top*) Venn diagrams showing the overlap between DIR genes (AD>CT) and DEPs downregulated in AD DLPFC from (*left*) combined sex and (*right*) female cohort. *P*-value was calculated by Fisher’s exact test. (*Bottom*) Enriched GO (Biological process) terms of the overlapping genes with increased IR and downregulated protein expression in AD DLPFC.

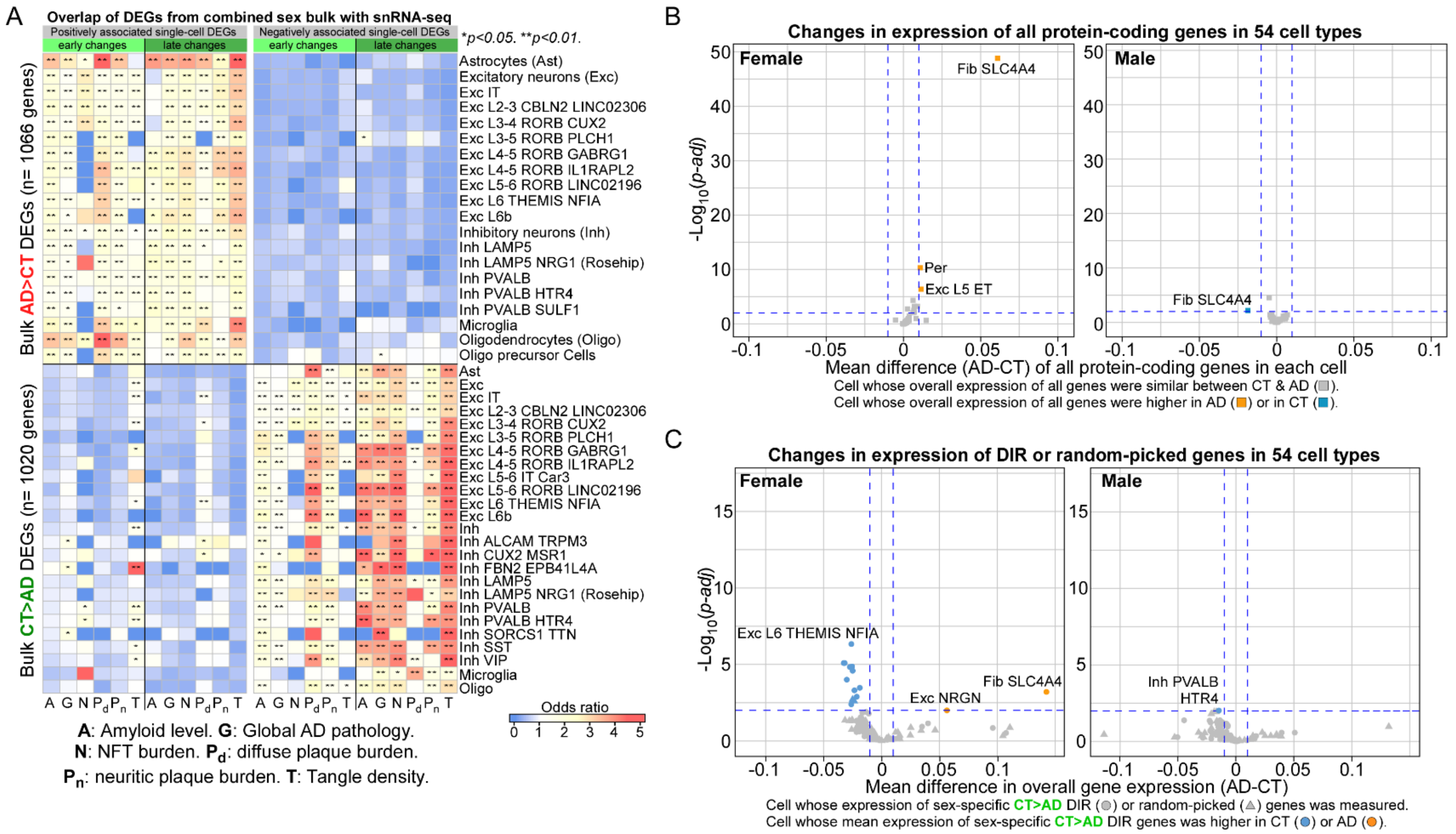

Figure S2. Comparison of bulk DEGs and DIR genes with single-cell DEGs

A) Heatmap showing the overlap of DEGs from combined sex bulk RNA-seq with DEGs from single-nucleus RNA-seq (snRNA-seq) of CT and AD PFC. The positively or negatively associated single-cell DEGs were classified according to early and late changes in the 5 different AD traits as defined by (Mathys et al., 2023). The overlap of bulk DEGs (AD>CT and CT>AD) with positively or negatively associated single-cell DEGs were determined independently to obtain top 20 cell types. Statistical significance was calculated by Fisher's exact test where $*p < 0.05$; $**p < 0.01$. The results indicated a high congruence between bulk and single-cell data such that AD>CT bulk DEGs were positively associated with AD traits whereas CT>AD DEGs were negatively associated with AD. Related to Figure 2A.

B) Volcano plot indicating expression changes (AD-CT) of all protein-coding genes between CT and AD in 54 cell types derived from female and male PFC. Each point is a single cell type defined by the mean difference and statistical significance of all the protein-coding genes between CT and AD. Paired *t*-test and Benjamini-Hochberg multiple corrections were performed with a cut-off of adjusted *p*-value < 0.01 (dotted blue horizontal line). Per, Exc L5 ET and Fib SLC4A4 cells have higher overall expression of all protein-coding genes in AD as compared to CT. Related to Figure 2A.

C) Volcano plot indicating expression changes (AD-CT) of the DIR (CT>AD) or random-picked genes between CT and AD in 54 cell types. Each point is a single cell type defined by the mean difference and statistical significance of either DIR (CT>AD) genes (circle) or matching numbers of random-picked genes (triangle). Paired *t*-test and Benjamini-Hochberg multiple corrections were performed with a cut-off of adjusted p-value < 0.01 (dotted blue horizontal line). Note that female DIR genes were compared to female snRNA-seq data while male DIR genes to male snRNA-seq data. Related to Figure 2A and 2B.

Figure S3

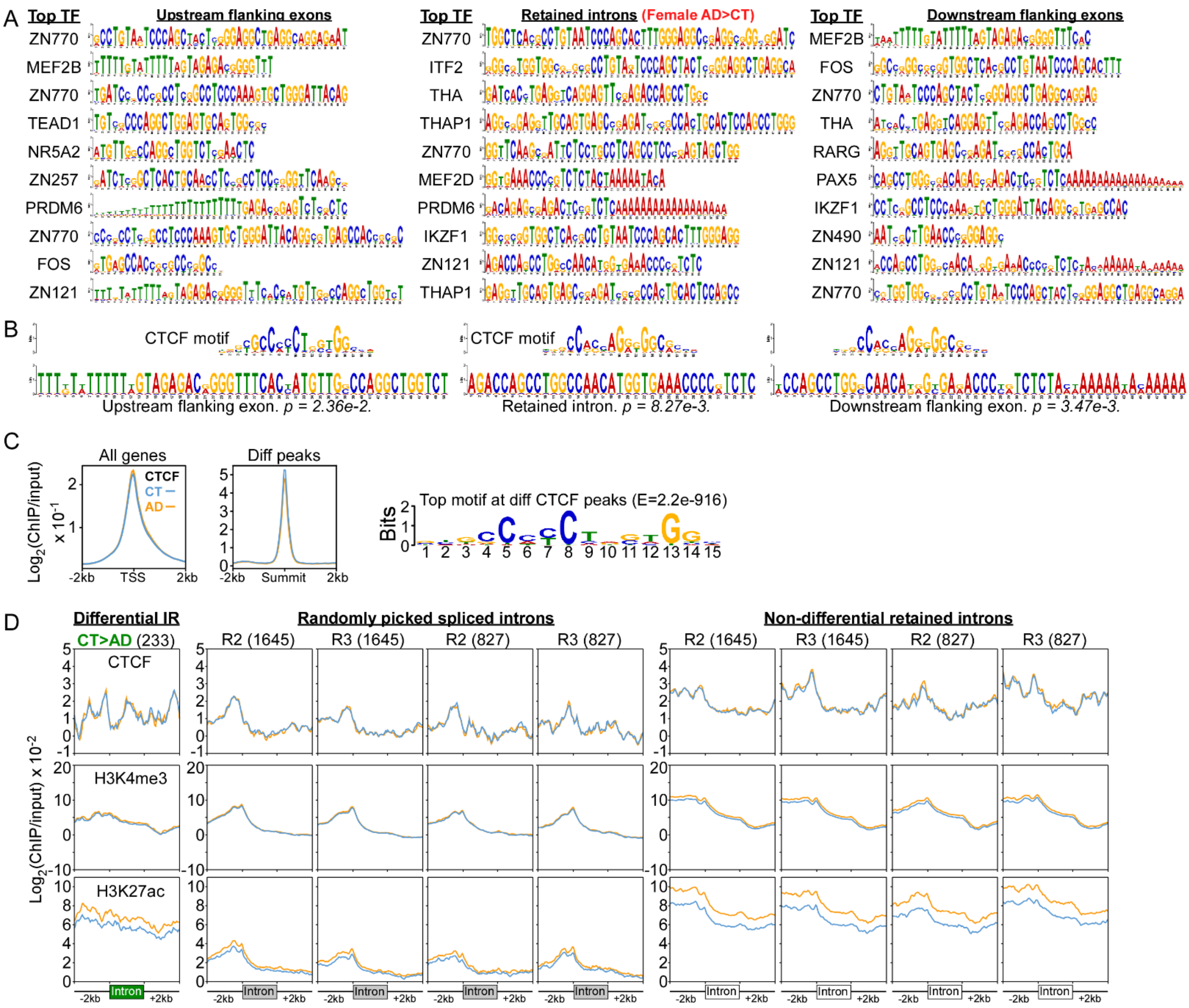

Figure S3. Enriched motifs and epigenetic profiles across different types of introns.

A) Top 10 DNA motifs enriched in introns differentially retained in female AD DLPFC (AD>CT) and their cognate transcription factor as predicted by TOMTOM.

B) Presence of CTCF binding motifs within the female differentially retained introns (AD>CT) (*middle*), their upstream (*left*) and downstream (*right*) flanking exons.

C) CTCF metaplot over (*left*) all genes and (*middle*) differential peaks defined by (Patel et al., 2023). (*Right*) Canonical CTCF motif is enriched at the differential CTCF peaks. TSS: Transcription start site.

D) Metaplots of CTCF, H3K4me3 and H3K27ac in female CT and AD DLPFC over the different introns and their flanking sequences (±2 kb). "R" stands for different replicates of random-picked introns. Parentheses indicate the number of different types of introns picked for metaplot analysis.

SUPPLEMENTAL METHODS

Rationale and aims of this study

Dorsolateral prefrontal cortex (DLPFC) plays important roles in cognitive control and executive function (Friedman and Robbins, 2022). In typical AD cases, amyloid-β deposition originates from frontal and temporal lobes (Masters et al., 2015) with pathological changes and dysfunction of DLPFC associated with AD progression (Kumar et al., 2017). As such, multi-omics studies have been conducted to elucidate mechanisms underlying sporadic AD pathology. DLPFC displayed sex-biased RNA and protein expression patterns (Guo et al., 2023; Wingo et al., 2023). Aberrant splicing events like differential intron retention (DIR) were also linked to AD (Adusumalli et al., 2019; Ngian et al., 2022; Raj et al., 2018). Combined sex analysis further revealed strong proteomic changes in AD that were not reflected at RNA level (Johnson et al., 2022). Given that women were more susceptible to AD (Guo et al., 2022), we aimed to address the following questions by performing detailed analysis of the available DLPFC datasets.

(i) Is there a sex-biased effect in the level of IR during AD progression?
(ii) What are the pathways affected by IR in each sex during AD progression?
(iii) Can DIR events explain the difference between RNA and proteomic changes in AD?
(iv) Is there a sex-biased difference in the AD-related proteomic changes?
(v) What are the possible mechanisms underlying differential IR in AD?

Transcriptomic and proteomic datasets

The FASTQ files downloaded were generated by RNA-seq of gray matter tissue from DLPFC (syn8612097) (De Jager et al., 2018; Raj et al., 2018) from the Religious Orders Study and Memory and Aging Project (ROSMAP) study cohorts (Bennett et al., 2012a; Bennett et al., 2012b). Metadata of all RNA-seq samples were provided in SD1a according to variables described in Rush Alzheimer's Disease Center codebook. Cases were classified based on (i) final clinical consensus diagnosis of cognitive status at the time of death (Cogdx) (Schneider et al., 2007), (ii) semiquantitative histopathological measures of neurofibrillary tangles with

Braak stage (braaksc) (Braak and Braak, 1991), and (iii) semiquantitative estimates of neuritic plaque density as recommended by the Consortium to Establish a Registry for AD (CERAD) scores (Mirra et al., 1991). The level of proteins in DLPFC (Brodmann area 9, BA9) from ROSMAP and the Banner Sun Health Research Institute study cohorts were quantified by tandem mass tag mass spectrometry (TMT-MS) approach (syn25006782) (Johnson et al., 2022) with metadata of samples used in this study listed in SD1b.

In both the ROSMAP and Banner cohorts, controls are defined with Braak stage ≤ 3 and CERAD scores = 3-4 whereas AD are defined with Braak stage ≥ 3 and CERAD scores = 1-2. Cognition measurement differs between the ROSMAP and Banner cohorts. For ROSMAP, controls have Cogdx = 1 while AD is defined by Cogdx = 4-5. Mini-Mental State Examination (MMSE) was used for Banner cohort (Folstein et al., 1975) where MMSE scores for controls are 25-30 and AD are ≤ 24.

For differential gene expression and intron retention analyses of RNA-seq dataset, there were 47 control and 121 AD female samples; as well as 39 control and 49 AD male samples (SD1a). For analysis of differential protein expression, there were 54 control and 108 AD female samples; as well as 52 control and 68 AD male samples following normalization/regression steps (SD1b).

Data processing and differential intron retention (IR) analysis.

The quality of downloaded FASTQ files was first evaluated by FastQC, followed by mapping to human genome (v107, GRCh38) with STAR aligner (v2.7.10a). Identification and quantification of differentially retained introns were performed by IRFinder-S software (v2.0.0) (Lorenzi et al., 2021) using FASTQ files as input in the following steps. First, the IR ratio was determined by dividing the abundance of intronic counts over the sum of intronic and exonic counts. Second, a minimum of 0.05 IR ratio in at least one group is needed to be qualified as a retained intron. Third, retained introns were discarded if 5% of samples triggered either a 'LowCover' warning, which indicates inadequate sequencing depth for the intron, or a 'LowSplicing' warning, which signals insufficient read support for proper splicing. Differential analysis of retained introns was executed using the 'IRFinder Diff' command with a statistical significance set at p-value < 0.05. The introns included in the analysis are exclusively from protein-coding genes (SD2).

Characterization of intronic length and GC content.

In-house scripts were utilized to extract intronic sequences for calculating their length and percentage of GC nucleotides. To control for the varied sample size between the differentially retained and spliced introns across different conditions, analyses were also performed on multiple randomly picked spliced introns that contain matching numbers as the test groups.

Visualization of IR levels against different AD traits

DESeq2 (Love et al., 2014) was used to generate normalized IR ratio counts which were then scaled and visualized with the ComplexHeatmap R package (Gu, 2022). Hierarchical clustering was applied to generate the heatmap for combined sex, female and male cohorts where each column is defined by the different AD traits such as Braak stage, Codgx and CERAD scores.

Identification of specific RBP binding motifs on retained introns.

Enrichment of specific RBP binding sites at either the differentially retained or spliced introns were determined using RBPmap software (version: v1.2) (Paz et al., 2014). The sequences of either full introns or exon-intron junctions (comprising of 300-bp intron and 50-bp exon) were retrieved and RBP-binding scores were calculated according to (Zhang et al., 2020) with minor modifications. First, introns were scanned for the presence of RBP-binding motif that occurs at least once within the sequence in the group (defined as binding frequency). Second, the total number of times each motif appeared was determined (defined as binding count). Third, RBP-binding score in each group was tabulated by the following formula to obtain top 20 motifs:

[(Binding frequency/number of introns) x (Binding count/number of introns)]/average length of introns in the group.

Following the removal of motifs with < 50% binding frequency in each group, up to 5 RBPs with the most significant *p*-values were assigned to each motif. Motifs identified from randomly picked spliced introns were used as background for subtraction. RBPs that are not unique and can be found in other categories have also been excluded.

Data processing and analysis of mRNA expression patterns.

To correct for batch effects, technical variations, and other biological differences between samples, RNA-seq counts (syn8691134) were subjected to regression with fixed effect model and workflow according to (Johnson et al., 2022; Sieberts et al., 2020). Genes with > 1 count per million total reads (CPM) in at least 50% of the samples were selected using CPM function in edgeR (Robinson et al., 2010), with additional filtering for genes with available gene length and GC content. Samples with incomplete metadata like RNA integrity number, post-mortem

interval, sex, or age at the time of death were excluded. The two diagnostic criteria in our study are control and AD cases.

Prior to normalization, sample outliers were identified through principal component analysis (PCA) and the aberrant log(CPM) distribution. Genes with more than 3 standard deviations of the aberrant distribution of the log(CPM) counts were assigned not applicable "NA" values. RNA-seq read counts were first subjected to conditional quantile normalization (CQN) to account for GC content and gene length variations. Then, the confidence of sampling abundance of raw CPM counts was estimated with a weighted linear model using voom-limma R package (Law et al., 2014).

Significant covariates (FDR < 0.1) in the data were identified by PCA. An iterative PCA approach using voom-limma package was performed to normalize and adjust the expression matrix. Covariates were added sequentially based on their significant associations with top PCs, excluding Diagnosis, Cogdx and *ApoE* alleles. In each iteration, the design matrix was constructed from residual covariates, and voom weights were computed for dispersion control. A linear model was fitted to the CQN expression based on the design matrix and voom weights. The process was repeated if significant residual covariates (FDR < 0.1) persisted.

To identify genes with significant differential expression, voom-limma was used for weighted linear modeling and contrast fitting while accounting for the identified covariates (FDR < 0.05). Sex was included as a covariate in combined sex analysis but not in gender-specific analysis.

Gene ontology (GO) analysis and overlap with curated AD genes.

GO terms (Biological Processes and Molecular Functions) enrichment analysis of identified gene clusters were performed with clusterProfiler R package (Yu et al., 2012). We excluded GO terms that have < 10 genes to reduce spurious enrichment due to chance. Within each group, we also removed GO terms with 'similarity index' of 0.7 to reduce redundancy. Curated AD genes were obtained from DisGeNET (Pinero et al., 2021) and the *p*-value of the overlap calculated by Pearson's Chi-squared test with Yates' continuity correction.

Processing and analysis of proteomic data.

The protein abundance data (syn25006782) were processed, normalized, and regressed for covariates according to the workflow described by (Johnson et al., 2022) with minor modifications.

TAMPOR (https://github.com/edammer/TAMPOR), a median polish procedure, was used to remove batch artifacts and batchwise variance from the proteomic abundance data. Proteins

with > 50% missing values were excluded from further analysis. TAMPOR applies a normalization equation that involves sample-wise and protein-wise median centering. This process, including log2 transformation and careful handling of missing values, results in a log2-transformed relative abundance matrix for proteomic data.

Outliers were detected by Z-transformed network connectivity metric, with a predefined threshold of |Z.k| > 3 standard deviations from the mean Z.k, and removed iteratively until no further detection. The consensus TMT protein abundance matrix for ROSMAP and Banner BA9 was subjected to the first round of non-parametric bootstrap regression. This regression entailed controlling for covariates such as age at death and postmortem interval multiplied by the median estimated coefficient obtained from 1,000 regression iterations for each protein. The regression incorporated uncensored ages at death, whereas diagnosis and sex were protected from regression. Following checking and the removal of new outliers, the case samples were subjected to a second round of bootstrap regression.

Differentially expressed proteins were determined by performing *t*-test between AD patients and healthy subjects with multiple testing correction using FDR adjustment ($p < 0.05$).

mRNA or protein expression of differential IR genes

Beeswarm Plot was used to visualize the mRNA and protein expression patterns of differential IR genes. To test the effect of sample size, the mRNA and protein expression patterns of either all genes or random picked fully spliced genes were also determined.

Motif Analysis of differentially retained introns

MEME Suite (v5.5.5) (Bailey et al., 2015) was used to determine the top 10 enriched DNA motifs within the differentially retained intron sequences and their flanking regions. TOMTOM tool was implemented to determine any significant match between the top 10 enriched DNA motifs and HOCOMOCO Human (v11 core) database, assigning only top three transcription factors based on E-values of the query motif.

Processing and integrative analysis of CTCF ChIP-seq data

CTCF, H3K4me3, and H3K27ac ChIP-seq data of female control and AD human DLPFC were downloaded from the Rush Alzheimer's Disease study performed by the ENCODE consortium (Consortium, 2012; Luo et al., 2020). ChIP-seq performed by Bradley Bernstein lab was subjected to single-end sequencing with metadata provided in SD1c to SD1e.

To account for the difference in sequencing depth among individual samples, BAM files were scaled to match the sample with lowest read counts using samtools (v1.16.1) (Danecek et al., 2021). Subsequently, the downscaled files were merged and log2 ratio of the ChIP over input was achieved using the bamCompare function in deepTools (v3.5.1) (Ramirez et al., 2016). In this step, the raw read signals were normalized using bins per million reads (BPM) with a bin size of 50 and the alignment BAM files were converted to bigwig signal files. The computeMatrix and plotHeatmap commands of deepTools were then employed to generate read density distribution plots for ChIP-seq signals over the different specific genomic regions of interest.

The following genomic regions were analyzed: all genes, published differential CTCF peaks between control and AD DLPFC (Patel et al., 2023), differentially retained introns, and matching numbers of randomly picked spliced or non-differentially retained introns.

Validation of IR by quantitative PCR

Total RNA from healthy control (R1234035-P, Lot#C404081) and AD brain (R1236035Alz-50, Lot#A507294) (AMSBIO) were purchased from BIOCHAIN. cDNA libraries were generated using high-capacity cDNA reverse transcription kit (Applied Biosystems) with 1 µg of total RNA pre-treated with DNase I (Thermo Scientific). Real-time quantitative PCR was carried out in triplicates (6 µl/reaction) with 0.2 µM of primers and 2X SYBR green master mix (Thermo Scientific) using 7900HT Fast Real-Time PCR machine (Applied Biosystems). Relative level of intron retention was calculated using $2^{-\Delta CT}$ method where threshold cycle (C_T) values were normalized to exon/exon primers pair (SD1f).

Survey expression of DIR genes in single-cell atlas generated from control and AD PFC

We used published single-cell atlas derived from prefrontal cortex (PFC) since their global AD-pathology-associated DEGs showed highly significant overlap with single-nucleus RNA-seq from DLPFC (Mathys et al., 2023). Single-cell DEGs positively and negatively associated with different AD stages and pathological traits across 54 cell types were downloaded from this link: https://github.com/mathyslab7/ROSMAP_snRNAseq_PFC/tree/main.

We first determined if AD-related gene expression changes identified from RNA-seq of bulk DLPFC tissues were observed at single-cell level. The odds ratio and statistical significance (*p*-value) of the overlap between bulk and single-nucleus RNA-seq DEGs was calculated by Fisher's exact test from GeneOverlap package (10.18129/B9.bioc.GeneOverlap) with heatmap generated by ComplexHeatmap package in R (Gu, 2022). The top 20 cell types whose

positively or negatively associated DEGs overlapped significantly with bulk RNA-seq DEGs were determined using the following criteria: (i) an overlap of at least 10 DEGs, (ii) the presence of > 100 single-cell DEGs, and (iii) p-value < 0.05. Data were presented in Fig.S2A and SD8.

We next surveyed the expression patterns of DIR genes in single-cell. The downloaded snRNA-seq data (syn52293417) were generated from 33 control and 65 AD female samples; as well as 39 control and 44 AD male samples. Data was processed using the Seurat package (v4.0) in R with sequencing depth corrected by log-normalization (Hao et al., 2024).

To determine any AD-related changes in gene expression patterns, the average mRNA level of individual gene in each cell type from control and AD condition was calculated. The difference in expression pattern was determined statistically by paired t-test and Benjamini-Hochberg multiple correction with false discovery rate (FDR) threshold set at adjusted p-value < 0.01. This analysis was performed for the DIR genes from each sex, with matching numbers of random-picked genes and all protein-coding genes as reference. Data were presented in Fig.2A-B, Fig.S2B-C and SD8.

DESCRIPTION OF Supplemental Data SD1 to SD9

https://drive.google.com/drive/folders/1GfHERCVCQ8iiOT7DKREetnCfH8G3PwOX?usp=sharing

SD1: Metadata, Quality control and primers list.

(a) Metadata for all RNA-seq samples used. (b) Metadata for proteomic samples used. (c-e) Metadata for ChIP-seq experiments in female CT and AD DLPFC with antibodies against (c) CTCF, (d) H3K4me3 and (e) H3K27ac marks. (f) Primer used for qPCR validation.

SD2: List of differential IR events between CT and AD.

(a-c) List of all and differential retained introns identified by IRFinder-S from (a) combined sex, (b) female and (c) male cohorts.

SD3: GO analysis of differential IR genes from combined sex and female cohorts.

(a-b) GO terms of (a) female and (b) combined sex differential IR (AD>CT) genes that overlapped with curated AD genes.

SD4: List of differentially expressed protein-coding genes.

(a-c) List of all genes and DEGs from (a) combined sex analysis, (b) female and (c) male cohorts.

SD5: GO terms of DEGs and statistical analysis of expression changes of DIR genes.

(a) GO terms of DEGs from combined sex analysis & (b) sex-specific DEGs. (c) P-values of gene expression patterns between CT & AD for all or differential IR (DIR) genes.

SD6: List of differentially expressed proteins & their overlap with M7/M42 modules.

(a-c) List of DEPs from (a) combined sex analysis, (b) female and (c) male cohorts.

(d) Overlap of DEPs with proteins in M7/M42 modules. (e) Unique genes or proteins with differential IR events. (f) P-values of protein expression patterns between CT & AD for all detected isoforms or those with differential IR mRNAs. (g) Overlap between DEPs & differential IR genes.

SD7: GO terms of differentially expressed proteins.

(a-b) GO terms of (a) CT > AD and (b) AD > CT DEPs that were common to both sexes.

(c-d) GO terms of male specific (c) CT > AD and (d) AD > CT DEPs.

(e-f) GO terms of female specific (e) CT > AD and (f) AD > CT DEPs.

(g-h) GO terms of CT > AD DEPs that overlapped with DIR (AD > CT) genes from (g) combined sex and (h) female cohorts.

SD8: Bulk vs snRNA-seq DEGs and DIR gene expression across 54 cell types in AD.

(a-b) Overlap of combined sex (A) AD > CT and (B) CT > AD DEGs from bulk DLPFC RNA-seq with single-nucleus RNA-seq data.

(c-d) Analysis of the changes in expression patterns (AD-CT) of genes of interest across 54 cell types derived from (c) female and (d) male CT & AD DLPFC.

SD9: RBP & DNA motifs enriched in differential retained introns.

(a-c) Top unique RBP motifs enriched in differential retained introns or 350 bases flanking exon-intron junctions from (A) combined sex analysis, (B) female and (C) male cohorts.

(d) Top 10 DNA motifs enriched in differential retained introns from female and combined sex cohorts with their predicted transcription factors.

REFERENCES

Adusumalli, S., Ngian, Z.K., Lin, W.Q., Benoukraf, T., and Ong, C.T. (2019). Increased intron retention is a post-transcriptional signature associated with progressive aging and Alzheimer's disease. Aging Cell *18*, e12928.

Bailey, T.L., Johnson, J., Grant, C.E., and Noble, W.S. (2015). The MEME Suite. Nucleic Acids Res *43*, W39-49.

Bennett, D.A., Schneider, J.A., Arvanitakis, Z., and Wilson, R.S. (2012a). Overview and findings from the religious orders study. Curr Alzheimer Res *9*, 628-645.

Bennett, D.A., Schneider, J.A., Buchman, A.S., Barnes, L.L., Boyle, P.A., and Wilson, R.S. (2012b). Overview and findings from the rush Memory and Aging Project. Curr Alzheimer Res *9*, 646-663.

Braak, H., and Braak, E. (1991). Neuropathological stageing of Alzheimer-related changes. Acta Neuropathol *82*, 239-259.

Consortium, E.P. (2012). An integrated encyclopedia of DNA elements in the human genome. Nature *489*, 57-74.

Danecek, P., Bonfield, J.K., Liddle, J., Marshall, J., Ohan, V., Pollard, M.O., Whitwham, A., Keane, T., McCarthy, S.A., Davies, R.M.*, et al.* (2021). Twelve years of SAMtools and BCFtools. Gigascience *10*.

De Jager, P.L., Ma, Y., McCabe, C., Xu, J., Vardarajan, B.N., Felsky, D., Klein, H.U., White, C.C., Peters, M.A., Lodgson, B.*, et al.* (2018). A multi-omic atlas of the human frontal cortex for aging and Alzheimer's disease research. Sci Data *5*, 180142.

Folstein, M.F., Folstein, S.E., and McHugh, P.R. (1975). "Mini-mental state". A practical method for grading the cognitive state of patients for the clinician. J Psychiatr Res *12*, 189-198.

Friedman, N.P., and Robbins, T.W. (2022). The role of prefrontal cortex in cognitive control and executive function. Neuropsychopharmacology *47*, 72-89.

Gu, Z. (2022). Complex heatmap visualization. Imeta *1*, e43.

Guo, L., Cao, J., Hou, J., Li, Y., Huang, M., Zhu, L., Zhang, L., Lee, Y., Duarte, M.L., Zhou, X.*, et al.* (2023). Sex specific molecular networks and key drivers of Alzheimer's disease. Mol Neurodegener *18*, 39.

Guo, L., Zhong, M.B., Zhang, L., Zhang, B., and Cai, D. (2022). Sex Differences in Alzheimer's Disease: Insights From the Multiomics Landscape. Biol Psychiatry *91*, 61-71.

Hao, Y., Stuart, T., Kowalski, M.H., Choudhary, S., Hoffman, P., Hartman, A., Srivastava, A., Molla, G., Madad, S., Fernandez-Granda, C.*, et al.* (2024). Dictionary learning for integrative, multimodal and scalable single-cell analysis. Nat Biotechnol *42*, 293-304.

Johnson, E.C.B., Carter, E.K., Dammer, E.B., Duong, D.M., Gerasimov, E.S., Liu, Y., Liu, J., Betarbet, R., Ping, L., Yin, L.*, et al.* (2022). Large-scale deep multi-layer analysis of Alzheimer's disease brain reveals strong proteomic disease-related changes not observed at the RNA level. Nat Neurosci *25*, 213-225.

Kumar, S., Zomorrodi, R., Ghazala, Z., Goodman, M.S., Blumberger, D.M., Cheam, A., Fischer, C., Daskalakis, Z.J., Mulsant, B.H., Pollock, B.G.*, et al.* (2017). Extent of Dorsolateral Prefrontal Cortex Plasticity and Its Association With Working Memory in Patients With Alzheimer Disease. JAMA Psychiatry *74*, 1266-1274.

Law, C.W., Chen, Y., Shi, W., and Smyth, G.K. (2014). voom: Precision weights unlock linear model analysis tools for RNA-seq read counts. Genome Biol *15*, R29.

Lorenzi, C., Barriere, S., Arnold, K., Luco, R.F., Oldfield, A.J., and Ritchie, W. (2021). IRFinder-S: a comprehensive suite to discover and explore intron retention. Genome Biol *22*, 307.

Love, M.I., Huber, W., and Anders, S. (2014). Moderated estimation of fold change and dispersion for RNA-seq data with DESeq2. Genome Biol *15*, 550.

Luo, Y., Hitz, B.C., Gabdank, I., Hilton, J.A., Kagda, M.S., Lam, B., Myers, Z., Sud, P., Jou, J., Lin, K.*, et al.* (2020). New developments on the Encyclopedia of DNA Elements (ENCODE) data portal. Nucleic Acids Res *48*, D882-D889.

Masters, C.L., Bateman, R., Blennow, K., Rowe, C.C., Sperling, R.A., and Cummings, J.L. (2015). Alzheimer's disease. Nat Rev Dis Primers *1*, 15056.

Mathys, H., Peng, Z., Boix, C.A., Victor, M.B., Leary, N., Babu, S., Abdelhady, G., Jiang, X., Ng, A.P., Ghafari, K., *et al.* (2023). Single-cell atlas reveals correlates of high cognitive function, dementia, and resilience to Alzheimer's disease pathology. Cell *186*, 4365-4385 e4327.
Mirra, S.S., Heyman, A., McKeel, D., Sumi, S.M., Crain, B.J., Brownlee, L.M., Vogel, F.S., Hughes, J.P., van Belle, G., and Berg, L. (1991). The Consortium to Establish a Registry for Alzheimer's Disease (CERAD). Part II. Standardization of the neuropathologic assessment of Alzheimer's disease. Neurology *41*, 479-486.
Ngian, Z.K., Tan, Y.Y., Choo, C.T., Lin, W.Q., Leow, C.Y., Mah, S.J., Lai, M.K., Chen, C.L., and Ong, C.T. (2022). Truncated Tau caused by intron retention is enriched in Alzheimer's disease cortex and exhibits altered biochemical properties. Proc Natl Acad Sci U S A *119*, e2204179119.
Patel, P.J., Ren, Y., and Yan, Z. (2023). Epigenomic analysis of Alzheimer's disease brains reveals diminished CTCF binding on genes involved in synaptic organization. Neurobiol Dis *184*, 106192.
Paz, I., Kosti, I., Ares, M., Jr., Cline, M., and Mandel-Gutfreund, Y. (2014). RBPmap: a web server for mapping binding sites of RNA-binding proteins. Nucleic Acids Res *42*, W361-367.
Pinero, J., Sauch, J., Sanz, F., and Furlong, L.I. (2021). The DisGeNET cytoscape app: Exploring and visualizing disease genomics data. Comput Struct Biotechnol J *19*, 2960-2967.
Raj, T., Li, Y.I., Wong, G., Humphrey, J., Wang, M., Ramdhani, S., Wang, Y.C., Ng, B., Gupta, I., Haroutunian, V., *et al.* (2018). Integrative transcriptome analyses of the aging brain implicate altered splicing in Alzheimer's disease susceptibility. Nat Genet *50*, 1584-1592.
Ramirez, F., Ryan, D.P., Gruning, B., Bhardwaj, V., Kilpert, F., Richter, A.S., Heyne, S., Dundar, F., and Manke, T. (2016). deepTools2: a next generation web server for deep-sequencing data analysis. Nucleic Acids Res *44*, W160-165.
Robinson, M.D., McCarthy, D.J., and Smyth, G.K. (2010). edgeR: a Bioconductor package for differential expression analysis of digital gene expression data. Bioinformatics *26*, 139-140.
Schneider, J.A., Arvanitakis, Z., Bang, W., and Bennett, D.A. (2007). Mixed brain pathologies account for most dementia cases in community-dwelling older persons. Neurology *69*, 2197-2204.
Sieberts, S.K., Perumal, T.M., Carrasquillo, M.M., Allen, M., Reddy, J.S., Hoffman, G.E., Dang, K.K., Calley, J., Ebert, P.J., Eddy, J., *et al.* (2020). Large eQTL meta-analysis reveals differing patterns between cerebral cortical and cerebellar brain regions. Sci Data *7*, 340.
Wingo, A.P., Liu, Y., Gerasimov, E.S., Vattathil, S.M., Liu, J., Cutler, D.J., Epstein, M.P., Blokland, G.A.M., Thambisetty, M., Troncoso, J.C., *et al.* (2023). Sex differences in brain protein expression and disease. Nat Med *29*, 2224-2232.
Yu, G., Wang, L.G., Han, Y., and He, Q.Y. (2012). clusterProfiler: an R package for comparing biological themes among gene clusters. OMICS *16*, 284-287.
Zhang, D., Hu, Q., Liu, X., Ji, Y., Chao, H.P., Liu, Y., Tracz, A., Kirk, J., Buonamici, S., Zhu, P., *et al.* (2020). Intron retention is a hallmark and spliceosome represents a therapeutic vulnerability in aggressive prostate cancer. Nat Commun *11*, 2089.
